# Effects of Ultrasonic Treatment on the Structure, Functional Properties of Chickpea Protein Isolate and Its Digestibility In Vitro

**DOI:** 10.3390/foods11060880

**Published:** 2022-03-19

**Authors:** Shihua Kang, Jian Zhang, Xiaobing Guo, Yongdong Lei, Mei Yang

**Affiliations:** School of Food Science and Technology, Shihezi University, Shihezi 832003, China; ksh20192011005@163.com (S.K.); zhangjian0411@163.com (J.Z.); leiyongdong1008@126.com (Y.L.); 20192111016@stu.shzu.edu.cn (M.Y.)

**Keywords:** ultrasonic treatment, chickpea protein isolate, chemical structure, functional properties, in vitro digestibility

## Abstract

This study evaluated the effects of different levels of ultrasonic power (200, 400, 600 W) and treatment time (0, 10, 15 and 30 min) on the structure, emulsification characteristics, and in vitro digestibility of chickpea protein isolate (CPI). The changes in surface hydrophobicity of CPI indicated that ultrasound treatment exposed more hydrophobic amino acid residues. The analysis of sulfhydryl content and zeta potential showed that ultrasound caused the disulfide bond of CPI to be opened, releasing more negatively charged groups, and the solution was more stable. In addition, Fourier Transform Infrared Spectroscopy (FT-IR) and intrinsic fluorescence spectroscopy showed that ultrasound changes the secondary and tertiary structure of CPI, which is due to molecular expansion and stretching, exposing internal hydrophobic groups. The emulsification and foaming stability of CPI were significantly improved after ultrasonic treatment. Ultrasonic treatment had a minor effect on the solubility, foaming capacity and in vitro digestibility of CPI. All the results revealed that the ultrasound was a promising way to improve the functional properties of CPI.

## 1. Introduction

Due to the increasing application of vegetable protein in food- and non-food-grade markets, the investigation of vegetable protein has attracted widespread attention in industrial production. Chickpeas (*Cicer arietinum* Linn.) are legumes of the genus *Chickpeas*, and are the second most consumed beans in the world and the third largest in the world in production. The protein content of CPI accounts for 15–30% of its total dry basis. Chickpea is a good source of protein with eight essential amino acids that are needed by the human body, especially lysine which is lacking in most grain proteins [1,2,3]. Chickpea protein isolate (CPI) is often investigated as an emulsifier, gelling agent and fat substitute in food due to its good functional properties [4]. However, due to the complexity of plant cell walls and the existence of various anti-nutritional factors, the actual application and production of chickpea protein is limited, and its nutritional value is affected by digestibility [5,6]. Therefore, it is necessary to seek different processing methods to improve chickpea protein to meet different functional and nutritional needs. At present, chemical methods and enzymatic methods have been used for the modification of chickpea protein, but there are disadvantages such as long operation time, high cost, large environmental pollution, and complicated follow-up processing [7]. Therefore, it is of great significance to explore green and efficient chickpea protein modification methods to improve its functional properties and expand its application in the food industry.

In recent years, ultrasound, as a new and promising technology, has been used for structural modification and functional optimization of biological macromolecules due to its green and efficient characteristics, especially for proteins [8,9,10,11]. The working mechanism of ultrasound is mainly the cavitation effect caused by the pressure difference in the ultrasound process, which makes the local energy density extremely high, which in turn produces micro-jet and turbulent motion. Ultrasound can achieve physical modification of protein through multiple effects such as cavitation, high shear, crushing and stirring. Compared with the traditional thermodynamic method, the ultrasonic technology reduces the processing cost with faster processing speed and better preserves the heat-unstable food ingredients, and also makes food processing implementation and operation more easier [12].

Protein has become an important emulsifier in food emulsions system due to its surface properties. The ability of protein to form and stabilize emulsions depends on the relative balance between its molecular size, charge, surface hydrophobicity and molecular flexibility. This study used chickpea protein as the research object, and measured the solubility, zeta potential, physicochemical properties, protein structure, in vitro digestibility, and emulsification properties of chickpea protein after different ultrasonic treatment conditions. In addition, the effects of ultrasonic treatment conditions on protein structure, functional properties, and in vitro digestibility of chickpeas were also discussed.

## 2. Materials and Methods

### 2.1. Materials and Reagents

The chickpeas used in the experiment were purchased from Ying Ge Biotechnology Co., Ltd., Mu lei, Xinjiang, China, and chickpeas were harvested in the current season. Pepsin (T4799) and trypsin (P7000) were purchased from Sigma-Aldrich. The reagents used in the experiment were of analytical grade. The water used in the experiment was deionized water.

### 2.2. Preparation of Chickpea Proteins

Chickpea proteins were extracted according to the method reported by Ghribi et al. with some modification [13]. Chickpea powder was defatted with n-hexane (1:3 [*w*/*v*]) for 4 h with a magnetic stirrer, repeated three times. 100 g defatted chickpea powder was mixed with water in the ratio of 1:10 (*w*/*v*). The pH value of defatted chickpea powder suspension was adjusted to 9.0 with 1 M NaOH solution, and then mechanically stirred at 500 rpm for 2 h at room temperature (25 °C). The mixture was centrifuged (4 °C, 5000× *g*, 15 min) to collect the supernatant. The precipitate was resuspended in water at a ratio of 1:5 (*w*/*v*), adjusted to pH 9.0, stirred at room temperature for 2 h, and centrifuged (5000× *g*, 15 min, 4 °C) to collect the supernatant. The supernatants were combined and adjusted to pH 4.5 with 0.1 M HCl to precipitate the protein. The precipitate was dissolved with water and then freeze-dried. The obtained chickpea protein isolate (CPI) was stored in a desiccator until further analysis. The protein, moisture, fat and ash content of CPI were 91.66% *w*/*w*, 4.50% *w*/*w*, 0.99% *w*/*w*, and 2.31% *w*/*w*, respectively.

### 2.3. Ultrasonic Treatment of CPI

Briefly, 6.0 g of CPI was dissolved in 100 mL distilled water and stirred at 25 °C for 2 h until fully hydrated. Then, the sample was sonicated in an ultrasound processor (SCIENTZ-ⅡD, Ningbo Xinzhi Biological Technology Co., Ltd. Ningbo, China) equipped with a 2.0 cm flat tip probe. The sample was treatment with different power (200 W, 400 W, or 600 W) and different time (10 min, 15 min, or 30 min). The turn-on and turn-off time of the ultrasound was 2 s. We performed an ice-water bath on the sample to keep the temperature of the sample at 25 ± 2 °C. The protein solution after ultrasonic treatment was freeze-dried and then stored at 4 °C. The untreated CPI was the control sample.

### 2.4. Solubility

A total of 1% (*w*/*v*) sample solution was prepared, and the protein content in the sample solution was measured by the biuret method. The samples were centrifuged (25 °C, 15 min, 10,000× *g*), and the supernatant was collected for absorbance measurement. Protein solubility is the ratio of the protein content in the supernatant to the total protein content in the CPI.

### 2.5. Zeta Potential

Zeta Potential of the samples was determined using Nano Particle Zeta Potential Analyzer (NanoPIUS-3, Mike Instruments, New York, NY, USA). It is measured under the condition of pH = 7 and protein concentration of 2% (*w*/*v*).

### 2.6. Physicochemical Characterization

#### 2.6.1. Surface Hydrophobicity

The surface hydrophobicity was determined through bromophenol blue (BPB) method that was reported by Chelh et al. [14]. The results were expressed as bound BPB (μg).

#### 2.6.2. Free and Total Sulfhydryl Groups

The free sulfhydryl and total sulfhydryl content in the sample were determined by the previous reported method with some modification [15].

Free sulfhydryl determination: The protein sample was dissolved in Tris buffer (0.1 M, pH 8.0) with 5.0% SDS at a concentration of 5 mg/mL. Then, the sample solution was heated at 80 °C for 45 min followed by centrifuged at 8000× *g* for 10 min. The supernatant was used for the determination of free sulfhydryl content.

Total sulfhydryl determination: Firstly, 0.3 mL β-mercaptoethanol (β-ME) was mixed with 1.8 mL sample solution (5 mg/mL, dissolved in SDS-Tris buffer) followed by incubated at 25 °C for 1 h. Secondly, 10 mL 12% TCA solution was added to that mixture, and continue to incubate for 1 h. Then, the incubated solution was centrifugated at 3000 rpm for 10 min. Subsequently, the precipitate was washed twice with 12% TCA. Finally, the washed precipitate was dissolved in the SDS-Tris buffer for the determination of total sulfhydryl content.

The above-mentioned solutions for sulfhydryl determination (0.75 mL) were mixed with 3.0 mL 5.0% SDS-Tris buffer followed by adding of 0.3 mL DTNB solution (10 mM). The solution absorbances were measured at 412 nm after incubation in the dark for 30 min. Formula (1) was used to calculate sulfhydryl content:

(1) $$C_{-SH}\;\left(\mu mol/g\right)=\frac{D\times 75.53\times A_{412}}{c}$$

*D* was the dilution factor, *c* was the weight of protein per volume (mg/mL), *A*_412_ was the absorbance of the solution at 412 nm.

### 2.7. Protein Structural Characterization

#### 2.7.1. SDS-PAGE

SDS-PAGE electrophoresis was performed at room temperature using a modified version of the method reported by Laemmli [16]. The concentration of CPI was adjusted to 2 mg/mL and mixed with loading buffer (with or without β-ME). The mixture was heated in a boiling water bath for 5 min. After centrifugation, the supernatants (10 μL) were added into the well of the gel. The gel consisted of 5% stacking gel and 12% separating gel. Electrophoresis was performed using the mini protean electrophoresis system (Bio-Rad Laboratories, Hercules, CA, USA) and the voltages were set to 80 V and 120 V, respectively. After completion of electrophoresis, the gel was stained with 0.25% Coomassie Brilliant Blue R-250 for 30 min. A 7.5% acetic acid, 25% methanol and a deionized-water-mixed solution was used for destaining. Untreated CPI solution was used as a negative control.

#### 2.7.2. Fourier Transform Infrared Spectroscopy (FT-IR)

The FT-IR information of samples were recorded by an FT-IR spectrometer (Bruker Vertex 70V, Shanghai, China) with scanning the whole band of (400–4000 cm^−1^) at room temperature [17].

#### 2.7.3. Intrinsic Fluorescence

The spatial conformation of chickpea protein was determined by fluorescence spectrometer [18]. The protein sample was dissolved in 10 mM phosphate-buffered solution (pH 7.0) with a concentration of 0.2 mg/mL. The test parameters of fluorescence spectrometer were set as follows: excitation wavelength 283 nm, slit width 5 nm, scanning speed 240 nm/min, and the fluorescence emission spectrum in the range of 300~450 nm was collected. The blank control was phosphate-buffered solution without chickpea protein.

### 2.8. Determination of CPI Emulsifying Properties

#### 2.8.1. Preparation of Emulsions

CPI solution (2% *w*/*v*) and corn oil were mixed in a ratio of 85:15 (*v*/*v*). Then, the mixtures of protein solution and corn oil were homogenized using a high-speed homogenizer at 13,000 r/min for 2 min to prepare emulsion.

#### 2.8.2. Emulsifying Activity Index (EAI) and Emulsifying Stability Index (ESI)

The emulsifying properties of CPI were determined by the method reported by Abir et al. [13]. In total, 25 μL emulsion (emulsion after homogenized 0 min or 10 min) was transferred into glass tube, then dilute it with 5 mL 0.1% sodium dodecyl sulfate solution. The absorbance of the diluted emulsions at 500 nm was measured on an ultraviolet spectrophotometer. Equations (2) and (3) were used to calculate emulsification activity index (*EAI*) and emulsification stability index (*ESI*):

(2) $$EAI\;\left(m^{2}/g\right)=\frac{2\times 2.303\times A_{0}\times \mathrm{DF}}{c\;\times \left(1-\phi \right)\times 10000}$$

(3) $$ESI\;\left(\min\right)=\frac{A_{0}}{A_{0}-A_{10}}\times 10$$

A_0_ was the absorbance of the coarse emulsion after 0 min of homogenization, DF was the dilution factor, c was the weight of protein per volume (g/mL), φ is the volume fraction of oil in the emulsion, and A_10_ is the absorbance of the emulsion after 10 min of homogenization.

### 2.9. Foaming Capacity (FC) and Foaming Stability (FS)

The protein sample was fully dissolved in distilled water, and 25 mL of protein solution was measured in a high foot beaker, and the homogenizer was used to stir at high speed for 1 min. The stirred solution was quickly transferred into the measuring cylinder. The volume of the foam was *V*_0_, and the *FC* of the protein was calculated by Formula (4):

(4) $$FC\left(\%\right)=\frac{V_{0}-25}{25}\times 100$$

*V*_20_ was the foam volume that was placed at room temperature for 20 min. The foam stability of the protein was calculated by Formula (5):

(5) $$FS\left(\%\right)=\frac{V_{20}-25}{V_{0}-25}\times 100$$

### 2.10. In Vitro Digestibility

The in vitro digestibility of CPI was conducted by Zhou et al. previous reports methods, with slight modification [19]. The protein sample was dissolved in distilled water (2% *w*/*v*) followed by the pH was adjusted to 1.6 with 1 M HCl. Then, pepsin (4% *w*/*w*, based on protein) was added. Afterwards, the mixture was incubated at 37 °C for 2 h. Subsequently, the pH of the solution was adjusted to 7.5 with 1 M NaOH, trypsin (4% *w*/*w*, based on protein) was added. The suspension was continuously incubated at 37 °C for 2 h, and then it was incubated in a boiling water bath for 10 min to stop the digestion. The gastrointestinal digestion product was obtained by centrifugation method (11,000× *g*, 15 min, 4 °C), and the supernatant was collected. The in vitro digestibility of CPI was expressed as the ratio of the protein content in the solution before and after digestion.

### 2.11. Statistical Analysis

One-way analysis of variance (ANOVA) was performed using IBM SPSS Statistics 25 software (SPSS Inc., Chicago, IL, USA). The results presented were the mean ± standard deviation, and the Duncan multiple range test analysis was used to compare the significance of the three-replicate data (*p* < 0.05).

## 3. Results

### 3.1. Solubility

Solubility is a key indicator of protein hydration that could significantly affect protein functional properties, and further determines protein applications [8]. As shown in Table 1, ultrasonic treatment enhanced the solubility of CPI, especially under the treatment conditions of 15 min and 600 W. Under the same ultrasonic power, the solubility increased with the increase in the ultrasonic time. Under the same ultrasonic time, the solubility firstly increased and then decreased with the increase in the ultrasonic power. These were due to that the cavitation and shear forces generated by ultrasound could depolymerize protein aggregates [20]. Therefore, the solubility of CPI was increased. However, excessive sonication would cause the protein to re-polymerize and reduce the solubility of the protein. Compared with the other treatment methods, the sonication treatment had a more direct effect on the protein solubility. Ultrasound could immediately generate high pressure that causes shear, cavitation, turbulence, surface static electricity and thermodynamics which would lead to protein denaturation, opening structure, and the surface being more negatively charged [21,22]. Hence, the sonication treatment could enhance the hydration of chickpea protein.

### 3.2. Zeta Potential

**Figure 1:**
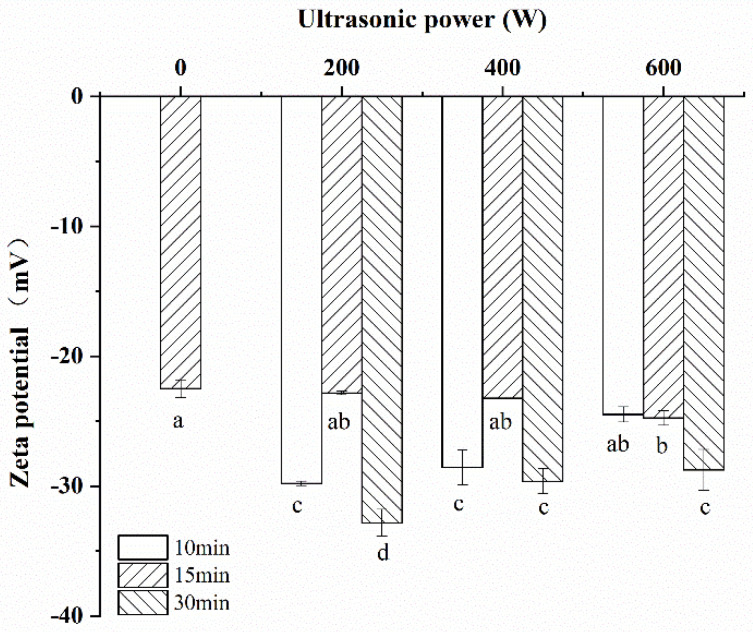
Zeta potential of CPI samples modified with different ultrasonic treatment conditions. ^a^^–^^d^ different letters in the same column indicate statistically significant differences at *p* < 0.05.

Zeta potential, the surface shear layer potential of charged particles in solution, is used to describe the electrostatic interactions between particles in solution, and its value is related to the suspended particle surface charge distribution [23]. As an important parameter, the surface charge of a particle not only affects its stability, but also affects the interaction with the other particles [24]. A larger absolute value of zeta potential indicates a stronger electrostatic interaction between protein molecules and its better dispersion stability in solution. As shown in Figure 1, after ultrasonic treatment, the absolute value of the zeta potential of CPI sample was higher than that sample without ultrasonic treatment. With the increase in the ultrasonic time, its absolute value shows a trend of first decrease and then increase. Those phenomena showed that ultrasonic treatment obviously increased the CPI′s degree of ionization (DI). This was probably due to the fact that more negatively charged groups of protein were exposed after ultrasonic treatment. However, its absolute value showed a decreasing trend when the sonication time was prolonged and/or the ultrasonic power was increased. The possible explanation for this trend was that the cavitation effect of ultrasound could cause the protein to re-aggregate which made the effective charge on the protein surface decreases.

### 3.3. Physicochemical Characterization

#### 3.3.1. Free and Total Sulfhydryl Groups

The disulfide bond is a covalent that was formed by the binding of the sulfhydryl groups of two cysteine residues through dihydroxylation. It is an important chemical bond that can stabilize the conformation of protein [25]. As shown in Table 1, with the prolongation of ultrasonic treatment time, the total sulfhydryl content of CPI showed a trend of first increase and then decrease. Interestingly, compared with untreated CPI, when the ultrasound time was 15 min, the total sulfhydryl content of CPI significantly increased, whereas the free sulfhydryl content obviously decreased. This was probably since the appropriate time of sonication exposed the sulfhydryl groups in the protein interior to its surface which could effectively promote the disulfide bond generation of CPI. However, the free sulfhydryl groups of the CPI significantly increased after sonication for 30 min. The possible explanation for this phenomenon was that prolonged ultrasound would further destroy disulfide bonds of CPI which caused the free sulfhydryl groups of protein to increase.

#### 3.3.2. Surface Hydrophobicity

Protein surface hydrophobicity, an important parameter, reflects the surface properties and the degree of deformation of the protein which can affect the functional properties of protein [26]. As shown in Table 1, when ultrasonic treatment time was the same, the CPI surface hydrophobicity showed a trend of increase first and then decrease with the increase in the ultrasonic power. This was because that shorter time or lower power cannot destroy the compact three-dimensional structure of the protein, leading to most of the hydrophobic amino acid residues which were still in the interior of the protein molecule. Although a longer time or higher power of ultrasonic treatment condition could increase the surface hydrophobicity of proteins. These were mainly due to the fact that the cavitation effect and mechanical stress generated by ultrasound destroyed the spatial structure of proteins leading to hydrophobic amino acids buried in the interior of proteins were exposed, resulting in the increase in protein surface hydrophobicity [10]. Whey protein concentrate, soy protein isolate, egg white protein and peanut protein isolate also showed similar results [26,27,28,29].

### 3.4. Protein Structural Characterization

#### 3.4.1. SDS-PAGE

**Figure 2:**
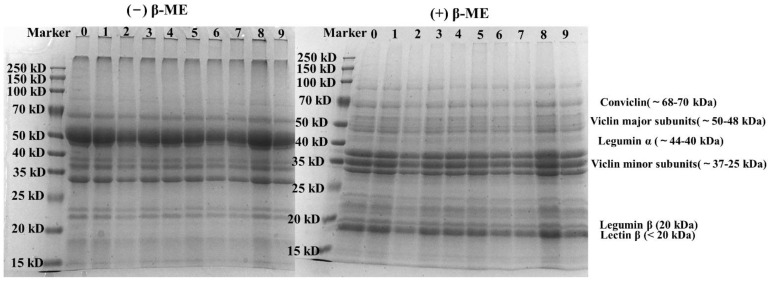
SDS-PAGE of CPI samples modified with different ultrasonic treatment conditions; 0-Unsonicated CPI; 1–3: 10 min sonication group (from 200 W to 600 W); 4–6: 15 min sonication group (from 200 W to 600 W); 7–9: 30 min sonication group (from 200 W to 600 W).

The molecular weight distribution of CPI (with or without ultrasonic treatment) was analyzed by non-reducing (-β-ME) and reducing (+β-ME) SDS-PAGE. As shown in Figure 2, CPI had clear bands at 20–25 kD and 32–72 kD. Regardless of whether SDS-PAGE was under reducing or non-reducing conditions, it can be observed that the molecular weight distribution of all samples was similar, which indicated that subunits of the CPI had not changed obviously after ultrasonic modification. This was because that ultrasonic treatment neither broke the original covalent bonds nor induced the formation of new ones [30]. These results were consistent with some reported researches [31].

#### 3.4.2. Secondary Structure

**Figure 3:**
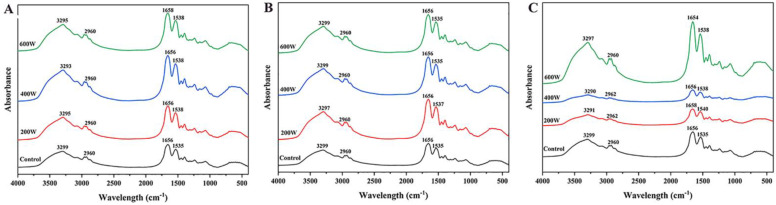
The effects of different ultrasonic treatment conditions on the FTIR of CPI samples. (**A**) 10 min, (**B**) 15 min, (**C**) 30 min.

FT-IR is usually used to evaluate the secondary structure of proteins. The amide I band (1700–1600 cm^−1^) of protein is mainly based on the C=O stretching vibration of the amide group (about 80%) which can be used to analyze the protein secondary structure [31,32]. As shown in Figure 3, the amide Ⅰ band of the CPI changed obviously after sonication indicating that the secondary structure of CPI was possibly changed. Hence, the spectrum of the amide I band of all CPI samples was analyzed by peakfit version 4.0 software. The 1648~1660 cm^−1^, 1626~1640 cm^−1^, 1662~1684 cm^−1^, and 1640~1650 cm^−1^ represented α-helix, β-sheet, β-turn, and random coil of protein, respectively [17,33]. Table 2 showed the content of each secondary structure of CPI samples. The contents of α-helix and random coils of CPI after ultrasonic treatment was significantly reduced (*p* < 0.05), whereas the content of β-structure (β-sheet and β-turn) was significantly increased (*p* < 0.05). The possible explanation for this result was that the cavitation and mechanical action produced by ultrasound destroyed the hydrogen bonds between the carbonyl group and the amino group on the polypeptide chain that stabilizes the α-helix structure, resulting in the content of α-helix decreasing and the content of β-structure increasing [26,28]. Under long-term sonication (30 min), more random coiled structures were transformed into β-sheet structures, which indicated that long-term sonication could promote protein cross-linking. Therefore, ultrasonic treatment was an effective method to modify protein secondary structure.

#### 3.4.3. Tertiary Structure—Intrinsic Fluorescence

**Figure 4:**
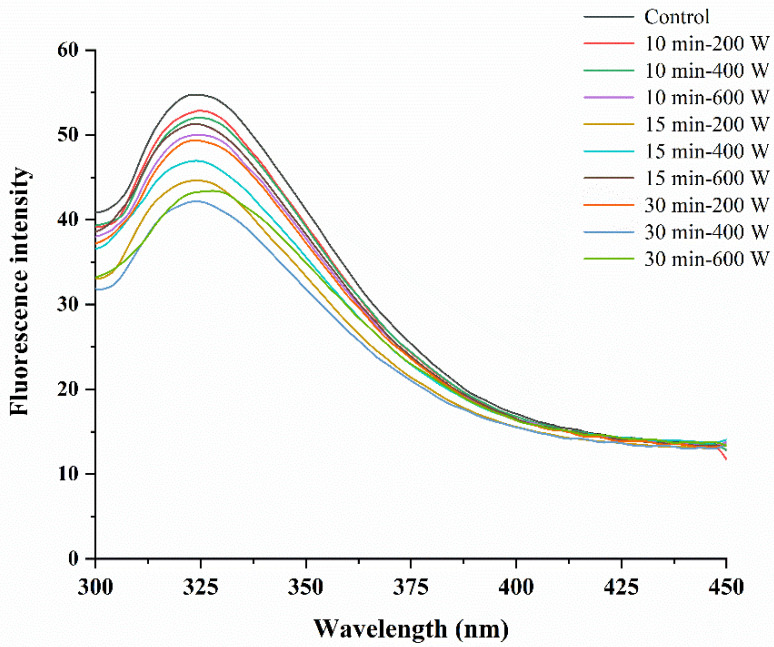
The intrinsic fluorescence spectrum of CPI samples modified with different ultrasonic treatment conditions.

Intrinsic fluorescence spectroscopy is an effective means to characterize the changes in the surface microenvironment of the fluorescent groups in protein molecules. When the excitation wavelength is at 280 nm, Tryptophan and tyrosine residues of protein which are exposed to the molecular surface have the fluorescence emission spectrum. Hence, the tertiary structure of the protein can be analyzed by the change of the fluorescence emission spectrum of fluorescent groups (tryptophan and tyrosine) [26]. As shown in Figure 4, compared with the control CPI, the fluorescence intensity of the ultrasonic-treated CPI was reduced obviously. This was probably due to the fact that the protein structure was destroyed by the ultrasound treatment, resulting in more fluorescent groups being exposed to the aqueous solution [10]. When the ultrasound treatment time was 10 min, the fluorescence intensity showed a decrease trend with the increase in the ultrasound power. Although the fluorescence intensity of CPI showed an increase trend with the increase in the ultrasound power when the ultrasound time was 15 min. This showed that a longer ultrasonic treatment time would re-form hydrophobic binding between protein molecules. Compared with the CPI without ultrasound treatment, the maximum emission wavelength of the CPI after ultrasound shifted to a longer wavelength, that is, a red shift. The red shift of the maximum emission wavelength further indicated that the tryptophan and tyrosine residues inside the protein were exposed to the aqueous solution after ultrasonic treatment [10,26,34].

### 3.5. Emulsifying Activity Index (EAI) and Emulsifying Stability Index (ESI)

**Figure 5:**
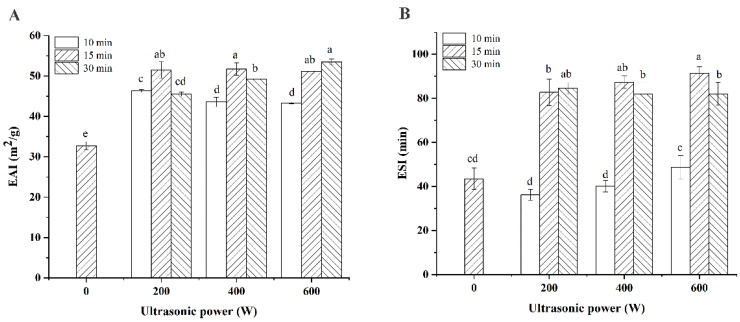
The EAI (**A**) and ESI (**B**) of different CPI samples (with or without ultrasonic treatment). ^a–e^ different letters in the same column indicate statistically significant differences at *p* < 0.05.

Protein has been widely used as an emulsifier in the food industry due to its good oil–water amphiphilic properties. Figure 5 showed the EAI and ESI of CPI with (or without) ultrasonic treatment. As shown in Figure 5A, it could be observed that ultrasound treatment significantly improved the EAI of CPI (*p* < 0.05). With the increase in sonication time, the EAI of CPI showed a variation trend of first increase and then decrease at low and medium powers (200 W, 400 W). These were because short-time, low-power sonication caused the protein molecules to partially unfold, meaning that the hydrophilic groups buried in the interior of the molecules were exposed, further resulting in elevation of the solubility of CPI, which enhanced the emulsification ability of the CPI. However, with the increase in sonication time, the protein deformation degree increased which led to the aggregation of molecules, so that the insoluble protein content increased, and the surface hydrophobicity decreased, which weakened its emulsification ability. However, under 600 W sonication power, the EAI of CPI showed an increasing trend with increasing sonication time. This was because the protein molecular structure became loose and disordered under high-power ultrasound which leaded to the emulsification capacity of CPI increased. Moreover, longer time or higher power ultrasonic treatment was beneficial to improve the ESI of CPI. Therefore, the cavitation effect produced during sonication treatment can enhance the flexibility of protein molecules through changing the interaction between protein molecules (such as hydrophobic interaction, hydrogen bonding and disulfide bonds) which improved the emulsifying properties of proteins [20,24,26,34].

### 3.6. Foaming Capacity (FC) and Foaming Stability (FS)

**Figure 6:**
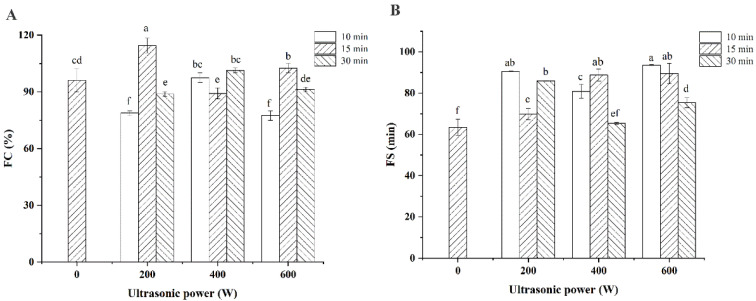
FC (**A**) and FS (**B**) of different CPI samples (with or without ultrasonic treatment). ^a^^–^^f^ different letters in the same column indicate statistically significant differences at *p* < 0.05.

Surface tension, surface hydrophobicity, particle size, and potential of proteins are important factors affecting protein blistering properties, and good protein blistering characteristics can confer good taste and soft structure to foods [35]. As shown in Figure 6A, ultrasound time and ultrasound power had minor effect on FC of CPI. When the ultrasonic treatment condition was 200 W/15 min, the modified CPI sample showed the best foaming ability. The possible explanation for this phenomenon was that the partial denaturation of proteins occurred under suitable modification condition leading to the exposure of hydrophobic regions and enhanced the adsorption of protein at the air–water interface, resulting in more foam [12,36]. However, with the intensification of ultrasonic treatment condition, the degree of protein denaturation increased inducing the formation of aggregates which leaded to the hydrophobic area of protein re-masked [37]. Hence, the adsorption capacity of the over-modified protein at the air–water interface was reduced, which worsened its foaming properties. Figure 6B showed the FS of different CPI samples. As shown in Figure 6B, the FS of CPI (*p* < 0.05) was significantly enhanced after ultrasonic modification. All the results indicated that ultrasonic treatment method was an effective method to improve the foaming properties of CPI.

### 3.7. In Vitro Digestibility

As shown in Table 1, the digestibility of CPI was affected by ultrasonic modification. In our study, the in vitro digestibility of untreated CPI was 89.28% which was consistent with the previous report [38]. When the ultrasonic treatment condition was relatively mild, the digestibility of modified CPI was less than that of control sample, which might be related to the change of CPI surface hydrophobicity [38]. The in vitro digestibility of CPI increased with the prolongation of ultrasonic time when the ultrasonic power was 200 W or 400 W. Although the ultrasonic power was 600 W, the digestibility of different CPI samples showed a tendency to first increase and then decrease. Moreover, the CPI sample modified under the condition of 600 W/15 min showed the highest digestibility. This was probably due to the fact that suitable ultrasonic treatment condition changed the protein conformation which could expose the cleavage sites of protein for digestive enzymes [38]. All the results indicated that suitable ultrasonic modification was beneficial to improve the digestibility of CPI.

## 4. Conclusions

In this study, ultrasound treatment had an effect on the structure, emulsifying properties, foaming properties, and in vitro digestibility of CPI. Among them, the effects on the emulsifying capacity, emulsifying stability, and foaming stability of CPI are significant, and the effects on the solubility, foamability, and in vitro digestibility are minor. After ultrasonic treatment, the changes in secondary and tertiary structure of CPI leaded to an increase in its surface hydrophobicity, total sulfhydryl content, and zeta potential which could affect the functional properties of CPI. When the ultrasonic treatment condition was 600 W/15 min, the modified CPI sample showed better emulsifying, foaming, and digestibility properties, and its EAI, ESI, FC, FS, and degree of digestibility were 51.16 m^2^/g, 91.46 min, 102.5%, 89.49%, 92.15%, respectively. Conversely, CPI performed poorly under sonication for 10 min. All the results indicated that ultrasonic processing technology was a promising way to improve the functional properties of CPI.

## Figures and Tables

**Table 1:** The solubility, surface hydrophobicity, free and total sulfhydryl groups, and in vitro digestibility of different CPI samples (with or without ultrasonic treatment).

Ultrasonic Treatment	Solubility/%	BPB Bound (μg)	Total-SH (μmol/g)	Free-SH (μmol/g)	In Vitro Digestibility (%)
Control	89.40 ± 2.69 ^cd^	12.96 ± 3.36 ^b^	59.95 ± 0.39 ^e^	9.52 ± 0.53 ^b^	89.28 ± 0.21 ^a^
10 min-200 W	93.66 ± 2.85 ^abcd^	6.70 ± 1.71 ^de^	55.84 ± 0.98 ^f^	9.77 ± 0.72 ^ab^	87.23 ± 0.51 ^fg^
10 min-400 W	96.46 ± 2.31 ^ab^	11.42 ± 1.02 ^bc^	50.93 ± 0.66 ^h^	9.87 ± 0.31 ^ab^	86.42 ± 0.73 ^gh^
10 min-600 W	93.09 ± 2.01 ^bcd^	13.76 ± 1.28 ^a^	53.25 ± 0.29 ^g^	9.34 ± 0.38 ^b^	85.41 ± 0.57 ^h^
15 min-200 W	92.85 ± 0.33 ^bcd^	9.05 ± 2.51 ^cd^	79.76 ± 0.64 ^a^	6.60 ± 0.55 ^d^	88.83 ± 0.09 ^e^
15 min-400 W	98.85 ± 2.47 ^a^	12.11 ± 0.24 ^b^	76.89 ± 0.79 ^b^	6.90 ± 0.11 ^d^	87.68 ± 0.21 ^f^
15 min-600 W	88.93 ± 3.16 ^d^	7.95 ± 1.04 ^de^	69.59 ± 0.62 ^c^	6.87 ± 0.40 ^d^	92.15 ± 0.64 ^de^
30 min-200 W	97.83 ± 5.19 ^ab^	5.70 ± 1.19 ^e^	62.31 ± 0.26 ^d^	10.52 ± 0.46 ^a^	90.21 ± 0.15 ^cd^
30 min-400 W	94.73 ± 3.52 ^abc^	5.29 ± 0.93 ^e^	60.51 ± 0.61 ^i^	9.06 ± 0.46 ^b^	90.47 ± 0.01 ^bc^
30 min-600 W	89.08 ± 0.97 ^cd^	13.94 ± 1.36 ^a^	59.12 ± 0.41 ^e^	8.21 ± 0.55 ^c^	91.36 ± 0.77 ^ab^

^a–h^ different letters in the same column indicate statistically significant differences at *p* < 0.05.

**Table 2:** The secondary structure of different CPI samples (with or without ultrasonic treatment).

Ultrasonic Treatment	α-Helix	β-Sheet	β-Turn	Random Coil
Control	25.49 ± 0.24 ^a^	21.71 ± 0.01 ^bc^	26.04 ± 0.04 ^d^	25.85 ± 0.08 ^a^
10 min-200 W	23.35 ± 0.30 ^d^	21.87 ± 0.22 ^abc^	27.64 ± 0.45 ^c^	24.06 ± 0.13 ^b^
10 min-400 W	24.75 ± 0.17 ^b^	22.10 ± 0.04 ^abc^	28.26 ± 0.11 ^bc^	23.63 ± 0.54 ^bc^
10 min-600 W	24.00 ± 0.02 ^bcd^	22.27 ± 0.11 ^abc^	28.22 ± 0.19 ^bc^	23.08 ± 0.37 ^bcd^
15 min-200 W	24.33 ± 0.19 ^bc^	21.38 ± 0.30 ^c^	28.05 ± 0.28 ^c^	22.50 ± 0.25 ^bc^
15 min-400 W	24.45 ± 0.17 ^bc^	22.38 ± 0.07 ^ab^	25.68 ± 0.05 ^d^	23.78 ± 0.01 ^bc^
15 min-600 W	24.54 ± 0.18 ^b^	22.70 ± 0.28 ^a^	25.92 ± 0.29 ^d^	24.51 ± 0.50 ^ab^
30 min-200 W	23.75 ± 0.18 ^cd^	21.55 ± 0.24 ^bc^	27.46 ± 0.48 ^c^	23.09 ± 0.45 ^bcd^
30 min-400 W	23.40 ± 0.03 ^d^	21.61 ± 0.59 ^bc^	29.77 ± 0.28 ^a^	22.21 ± 0.81 ^cd^
30 min-600 W	23.39 ± 0.70 ^d^	21.69 ± 0.76 ^bc^	29.47 ± 0.38 ^ab^	21.55 ± 0.52 ^d^

^a^^–^^d^ different letters in the same column indicate statistically significant differences at *p* < 0.05.

## Data Availability

The data presented in this study are available on request from the corresponding author.

## References

[1] Xu Y.X., Obielodan M., Sismour E., Arnett A., Alzahrani S., Zhang B.S. (2017). Physicochemical, functional, thermal and structural properties of isolated Kabuli chickpea proteins as affected by processing approaches. Int. J. Food Sci. Technol..

[2] Xu Y.X., Cartier A., Obielodan M., Jordan K., Hairston T., Shannon A., Sismour E. (2016). Nutritional and anti-nutritional composition, and in vitro protein digestibility of Kabuli chickpea (Cicer arietinum L.) as affected by differential processing methods. J. Food Meas. Charact..

[3] Ghribi A.M., Gafsi I.M., Blecker C., Danthine S., Attia H., Besbes S. (2015). Effect of drying methods on physico-chemical and functional properties of chickpea protein concentrates. J. Food Eng..

[4] Ladjal-Ettoumi Y., Boudries H., Chibane M., Romero A. (2016). Pea, Chickpea and Lentil Protein Isolates: Physicochemical Characterization and Emulsifying Propertiesu. Food Biophys..

[5] Serrano-Sandoval S.N., Guardado-Felix D., Gutierrez-Uribe J.A. (2019). Changes in digestibility of proteins from chickpeas (Cicer arietinum L.) germinated in presence of selenium and antioxidant capacity of hydrolysates. Food Chem..

[6] Bessada S.M.F., Barreira J.C.M., Oliveira M.B.P.P. (2019). Pulses and food security: Dietary protein, digestibility, bioactive and functional properties. Trends Food Sci. Technol..

[7] Felix M., Isurralde N., Romero A., Guerrero A. (2018). Influence of pH value on microstructure of oil-in-water emulsions stabilized by chickpea protein flour. Food Sci. Technol. Int..

[8] Higuera-Barraza O.A., Del Toro-Sanchez C.L., Ruiz-Cruz S., Marquez-Rios E. (2016). Effects of high-energy ultrasound on the functional properties of proteins. Ultrason. Sonochem..

[9] Jiang L., Wang J., Li Y., Wang Z., Liang J., Wang R., Chen Y., Ma W., Qi B., Zhang M. (2014). Effects of ultrasound on the structure and physical properties of black bean protein isolates. Food Res. Int..

[10] Jin J., Okagu O.D., Yagoub A.E.A., Udenigwe C.C. (2021). Effects of sonication on the in vitro digestibility and structural properties of buckwheat protein isolates. Ultrason. Sonochem..

[11] Flores-Jimenez N.T., Armando Ulloa J., Urias Silvas J.E., Ramirez Ramirez J.C., Rosas Ulloa P., Bautista Rosales P.U., Carrillo Y.S., Leyva R.G. (2019). Effect of high-intensity ultrasound on the compositional, physicochemical, biochemical, functional and structural properties of canola (Brassica napus L.) protein isolate. Food Res. Int..

[12] Tellez-Morales J.A., Hernandez-Santo B., Rodriguez-Miranda J. (2020). Effect of ultrasound on the techno-functional properties of food components/ingredients: A review. Ultrason. Sonochem..

[13] Ghribi A.M., Gafsi I.M., Sila A., Blecker C., Danthine S., Attia H., Bougatef A., Besbes S. (2015). Effects of enzymatic hydrolysis on conformational and functional properties of chickpea protein isolate. Food Chem..

[14] Chelh I., Gatellier P., Sante-Lhoutellier V. (2006). Technical note: A simplified procedure for myofibril hydrophobicity determination. Meat Sci..

[15] Shimada K., Cheftel J.C. (1988). Determination of sulfhydryl groups and disulfide bonds in heat-induced gels of soy protein isolate. J. Agri. Food Chem..

[16] Laemmli U.K. (1970). Cleavage of structural proteins during the assembly of the head of bacteriophage T4. Nature.

[17] Kang D.-C., Zou Y.-H., Cheng Y.-P., Xing L.-J., Zhou G.-H., Zhang W.-G. (2016). Effects of power ultrasound on oxidation and structure of beef proteins during curing processing. Ultrason. Sonochem..

[18] Cai L., Zhang W., Cao A., Cao M., Li J. (2019). Effects of ultrasonics combined with far infrared or microwave thawing on protein denaturation and moisture migration of Sciaenops ocellatus (red drum). Ultrason. Sonochem..

[19] Zhou F., Zhao M., Cui C., Sun W. (2015). Influence of linoleic acid-induced oxidative modifications on physicochemical changes and in vitro digestibility of porcine myofibrillar proteins. LWT.

[20] Yan S., Xu J., Zhang S., Li Y. (2021). Effects of flexibility and surface hydrophobicity on emulsifying properties: Ultrasound-treated soybean protein isolate. LWT.

[21] Alavi F., Chen L., Emam-Djomeh Z. (2021). Effect of ultrasound-assisted alkaline treatment on functional property modifications of faba bean protein. Food Chem..

[22] Jambrak A.R., Mason T.J., Lelas V., Herceg Z., Herceg I.L. (2008). Effect of ultrasound treatment on solubility and foaming properties of whey protein suspensions. J. Food Eng..

[23] Akbari A., Wu J. (2016). Cruciferin nanoparticles: Preparation, characterization and their potential application in delivery of bioactive compounds. Food Hydrocoll..

[24] Shen X., Fang T., Gao F., Guo M. (2017). Effects of ultrasound treatment on physicochemical and emulsifying properties of whey proteins pre- and post-thermal aggregation. Food Hydrocoll..

[25] Zhang Q.-T., Tu Z.-C., Xiao H., Wang H., Huang X.-Q., Liu G.-X., Liu C.-M., Shi Y., Fan L.-L., Lin D.-R. (2014). Influence of ultrasonic treatment on the structure and emulsifying properties of peanut protein isolate. Food Bioprod. Process..

[26] Chen L., Chen J., Ren J., Zhao M. (2011). Effects of Ultrasound Pretreatment on the Enzymatic Hydrolysis of Soy Protein Isolates and on the Emulsifying Properties of Hydrolysates. J. Agri. Food Chem..

[27] Hu H., Wu J., Li-Chan E.C.Y., Zhu L., Zhang F., Xu X., Fan G., Wang L., Huang X., Pan S. (2013). Effects of ultrasound on structural and physical properties of soy protein isolate (SPI) dispersions. Food Hydrocoll..

[28] Arzeni C., Martinez K., Zema P., Arias A., Perez O.E., Pilosof A.M.R. (2012). Comparative study of high intensity ultrasound effects on food proteins functionality. J. Food Eng..

[29] Zhu Z., Zhu W., Yi J., Liu N., Cao Y., Lu J., Decker E.A., McClements D.J. (2018). Effects of sonication on the physicochemical and functional properties of walnut protein isolate. Food Res. Int..

[30] Deng X., Ma Y., Lei Y., Zhu X., Zhang L., Hu L., Lu S., Guo X., Zhang J. (2021). Ultrasonic structural modification of myofibrillar proteins from Coregonus peled improves emulsification properties. Ultrason. Sonochem..

[31] Liu L., Zeng J., Sun B., Zhang N., He Y., Shi Y., Zhu X. (2020). Ultrasound-Assisted Mild Heating Treatment Improves the Emulsifying Properties of 11S Globulins. Molecules.

[32] Jackson M., Mantsch H.H. (1995). The use and misuse of FTIR spectroscopy in the determination of protein-structure. Crit. Rev. Biochem. Mol. Biol..

[33] Li L., Zhou Y., Teng F., Zhang S., Qi B., Wu C., Tian T., Wang Z., Li Y. (2020). Application of ultrasound treatment for modulating the structural, functional and rheological properties of black bean protein isolates. Int. J. Food Sci. Technol..

[34] O'Sullivan J., Murray B., Flynn C., Norton I. (2016). The effect of ultrasound treatment on the structural, physical and emulsifying properties of animal and vegetable proteins. Food Hydrocoll..

[35] Xiong T., Xiong W., Ge M., Xia J., Li B., Chen Y. (2018). Effect of high intensity ultrasound on structure and foaming properties of pea protein isolate. Food Res. Int..

[36] Malik M.A., Sharma H.K., Saini C.S. (2017). High intensity ultrasound treatment of protein isolate extracted from dephenolized sunflower meal: Effect on physicochemical and functional properties. Ultrason. Sonochem..

[37] Xiong W., Wang Y., Zhang C., Wan J., Shah B.R., Pei Y., Zhou B., Li J., Li B. (2016). High intensity ultrasound modified ovalbumin: Structure, interface and gelation properties. Ultrason. Sonochem..

[38] Wang X., Gao W., Zhang J., Zhang H., Li J., He X., Ma H. (2010). Subunit, amino acid composition and in vitro digestibility of protein isolates from Chinese kabuli and desi chickpea (Cicer arietinum L.) cultivars. Food Res. Int..

